# Comparative efficacy of pharmacological and non-pharmacological interventions for the acute treatment of adult outpatients with anorexia nervosa: study protocol for the systematic review and network meta-analysis of individual data

**DOI:** 10.1186/s40337-017-0153-3

**Published:** 2017-08-02

**Authors:** Tracey D. Wade, Janet Treasure, Ulrike Schmidt, Christopher G. Fairburn, Susan Byrne, Stephan Zipfel, Andrea Cipriani

**Affiliations:** 1 School of Psychology, Flinders University, PO Box 2100, Adelaide, SA 5001 Australia; 2 Department of Psychological Medicine, Kings College London, London, UK; 3 Department of Psychiatry, Oxford University, Oxford, UK; 4 School of Psychology, University of Western Australia, Perth, Australia; 5 Department of Internal Medicine (Psychosomatic Medicine and Psychotherapy), University Medical Hospital Tuebingen, Tübingen, Germany

**Keywords:** Network meta-analysis, Anorexia nervosa, Outpatient treatment

## Abstract

**Background:**

Outpatient treatment studies of anorexia nervosa (AN) are notoriously hard to conduct given the ambivalence of the patient group and high drop-out rates. It is therefore not surprising that previous meta-analyses of pharmacological and psychological treatments for outpatient treatment of adult AN have proved to be inconclusive. Network meta-analysis (NMA) has the potential to overcome the limitations of pairwise meta-analysis, as this approach can compare multiple treatments using both direct comparisons of interventions within randomized controlled trials (RCTs) and indirect comparisons across trials based on a common comparator. To date there is no published example of this approach with eating disorders and the current study provides a protocol which will use NMA to advance knowledge about what outpatient therapy works best for which patients with AN by conducting both direct and indirect comparisons of different treatments and the moderating variables.

**Methods:**

Searches of electronic data bases will be supplemented with manual searches for published, unpublished and ongoing RCTs in international registries, and clinical trials registries of regulatory agencies and pharmaceutical companies. Two reviewers will independently extract the data and where possible we will access individual data in order to examine moderators of treatment. Two primary outcomes will be selected: changes to body mass index and changes to global eating disorder psychopathology. The secondary outcome is the total number of patients who, at 12-month post-randomization, attained over the previous 28 day period: (i) BMI > 18.5, and (ii) global eating disorder psychopathology to within 1 SD of community norms. We will also provide a statistical evaluation of consistency, the agreement between direct and indirect evidence.

**Results:**

Descriptive statistics across all eligible trials will be provided along with a network diagram, where the size of the nodes will reflect the amount of evidence accumulated for each treatment. We will use a contribution matrix that describes the percentage contribution of each direct meta-analysis to the entire body of evidence.

**Discussion:**

Findings will make a major contribution to the literature by summarising individual data across rapidly accumulating outpatient trials of AN using state of the art NMA methodology.

**Trial registration:**

PROSPERO registration number: CRD42017064429

## Background

Anorexia nervosa (AN) is an eating disorder characterised by behavioural disturbance related to eating or weight control practices that leads to a significantly low body weight, a disturbance in the experience of body shape and/or weight, and a significant impairment in physical, social, vocational and psychological functioning. The restricting subtype involves energy restriction, increased energy expenditure, fasting and other non-purging compensatory behaviours in the absence of binge eating; the binge-eating/purging type includes the presence of binge eating or purging behaviours (or both). The proposed ICD-11 and DSM-5 criteria for AN are similar, including the weight specification and removal of the requirement of amenorrhoea that was present in previous systems of diagnosis.

While severe AN is typically treated in an inpatient or residential environment using multimodal treatment delivered by multidisciplinary teams [1], there is consensus that outpatient psychotherapy is required in addition to specialist care that includes nutritional rehabilitation and weight restoration in order to achieve recovery [2]. While there is evidence for the benefit of including parents in outpatient treatments for young people (i.e., < 18 years) [3–5], no specific outpatient psychotherapy has shown superiority for adults with AN [1]. A meta-analysis of 57 psychological treatment studies was inconclusive [6], yielding no salient results supporting a particular therapy technique, setting or procedure. Additionally, there is no clarity on the most effective pharmacotherapy for this disorder. In a meta-analysis of a small number of pharmacotherapy studies, pooled effect sizes of the difference between placebo and both antidepressants and antipsychotics on weight were not significant [7]. Hormonal therapy had a significantly larger effect on weight compared to placebo but heterogeneity was high, indicating caution with respect to interpretation of the results.

In part, these inconclusive findings are explained by a lack of power. Given the high mortality rate associated with AN, the use of non-active comparator conditions is rare, and there are relatively small numbers of participants in any given study due to the ambivalence of this client group to receive and remain in treatment [8]. This also means that treatment studies have not been able to sufficiently address the more complex treatment approaches seen in routine clinical practice [1], such as multidisciplinary treatment or adjunctive use of hospital admission for physical safety.

However the number of studies investigating outpatient treatment approaches for AN is rapidly increasing. For example, in 2013 there were 40 randomised controlled trials (RCTs) in progress around the same amount of trials that had emerged over a 30 year period between 1981 and 2013 [1]. Given the last meta-analysis of psychological treatments was conducted in 2011 and the results of the 40 RCTs have not yet been captured in systematic reviews, it is now timely to consider another meta-analysis. In particular, network meta-analysis (NMA) is an ideal tool to apply to such an area in terms of advancing knowledge about what works best for whom as it is a statistical technique that allows both direct and indirect comparisons to be undertaken, even when pairs of the treatments have not been compared directly (head to head) in the same trial [9, 10]. Of the 40 studies mentioned above, only 9 (23%) directly compared different types of therapy (as opposed to studies which compare medications, or forms of cognitive behaviour therapy [CBT] or forms of family therapy). Traditional meta-analyses would be unable to provide direct comparisons but NMA can provide point estimates of relative efficacy between all interventions even though some have never been compared head to head, as well as an estimate of inconsistency e.g., how well the entire network fits together. NMA has already been used successfully in other fields of psychiatry [11].

However, in the field of AN there is heterogeneity in treatment effects and many factors can have an impact on the relative treatment. In order to identify the prognostic factors (variables that predict overall response regardless of the treatments) and effect modifiers (variables that predict differential response to alternative treatments), the best methodological approach is to apply meta-regression to the NMA of individual participant data (IPD-NMA) [12]. It will enable a more powerful examination of the influence of both group-level and individual-level characteristics on the outcomes in the comparison of three or more alternative treatments [9]. The objective of the proposed systematic review and NMA is to compare acute phase monotherapy treatments of AN in terms of efficacy and acceptability in order to better inform clinical practice and mental health policies at the individual patient level.

## Methods/Design

### Criteria for considering studies for this review

#### Types of studies

Given the challenges inherent in conducting large treatment studies for AN and the need to increase our power as far as possible, only RCTs (either double-blind, single-blind or non-blind reported as comparing one active outpatient condition with another, or with wait list) offering acute phase treatment of AN will be included. Acute phase of illness is defined as treatment with participants who currently meet criteria for AN; this can be contrasted to maintenance therapy, where participants are randomised to therapy arms after weight restoration [1]. Only monotherapy studies will be included, defined as treatments that were not primarily used as an augmentation strategy (e.g., to an inpatient or outpatient treatment, or as maintenance of weight gain after a lengthy inpatient admission). Given that weight loss is central to the psychopathology of AN, individuals will be included if they had to have short inpatient stays for the purpose of medical stabilisation in order to enable them to participate in the main curative therapy (either before therapy commences or during therapy. Across studies, the use of short-term hospitalisation is commonly incorporated into the analyses. For example, the Anorexia Nervosa Treatment of Outpatients study incorporated intermittent inpatient treatment of up to 4 weeks in order to enable a BMI > 15 to be attained as required by their ethics approval process for outpatient treatment [13]. In two different comparisons of specialist supportive clinical management and the Maudsley Model of Anorexia Nervosa Treatment for Adults (MANTRA), days of hospital care were recorded and compared between the groups [14, 15].

#### Types of participants

Patients aged 18 years or older, of both sexes, with a primary diagnosis of AN, will be included. Studies adopting any standard operationalised diagnostic criteria to define patients suffering from anorexia nervosa will be included e.g., DSM-III, DSM-IIIR, DSM-IV, DSM-5 and ICD-10. Studies using clinical cut-off scores on symptom rating scales to indicate a potential disorder will not be included, and neither will studies that rely on participant self-report, but where participants self-report past 12 month diagnosis of AN from a doctor, or past 12 month treatment for AN (including hospital admission), these will be included.

Given that the presence of other psychiatric disorders are common (e.g., depression and anxiety disorders), and their status as primary or secondary diagnoses is unclear until the AN is treated, all concurrent diagnoses will not be considered as an exclusion criterion, but will be recorded for examination as a moderating factor. Patients will need to have completed the intensive form of the treatment to be included in this NMA but may still be receiving some type of maintenance support, including medications.

#### Types of interventions

We are interested in comparing therapeutic modalities that have the *aim* of achieving remission during the acute phase of illness. Definitions of remission typically include *both* body mass index (BMI) and eating disorder psychopathology, as it is not uncommon for treatment to lead to improvement in one but not both [e.g., 14], where the patient is still considered to have significant impairment. The specific modalities to be investigated include the following: antidepressants, antipsychotics, hormonal treatment, CBT, behavioural therapy, treatment as usual (TAU), cognitive analytic therapy, focal psychodynamic psychotherapy (FPT), interpersonal psychotherapy, specialist supportive clinical management (formerly non-specific supportive clinical management), ego-oriented individual therapy/adolescent-focused individual therapy, body awareness therapy, family-based treatment/family therapy, MANTRA, cognitive interpersonal treatment, cognitive remediation therapy, Loughborough Eating disorders Activity therapy, exposure and response prevention, and psychoanalytic psychotherapy.

### Search strategy and study selection

The following electronic databases will be searched: CENTRAL, CINAHL, MEDLINE, MEDLINE In-Process and PSYCINFO. The electronic searches will be supplemented with manual searches for published, unpublished and ongoing RCTs in international registries (such as clinicaltrials.gov), and clinical trials registries of regulatory agencies and pharmaceutical companies (see Appendix for the full list of resources). It is important to include unpublished data, since publication bias leads to exaggerated effect sizes and reporting bias can bias NMA-based estimates of treatments efficacy and modify ranking [16]. Studies will be identified using Keywords anorexia* (abstract) and treat* (abstract). No data limits or language restrictions will be applied to any of the searches. The reference lists of included studies will be searched for additional studies. Where eligible studies are found, supplemental data will be requested from the investigators if needed. We will include all studies, irrespective of their country of origin, identified in the international databases listed above and satisfying our eligibility criteria. Two persons will independently select references and abstracts retrieved by the search. If both reviewers agree that a trial does not meet eligibility criteria, it will be excluded. We will obtain the full text of all remaining articles and use the same eligibility criteria to determine which, if any, to exclude at this stage. Any disagreements will be resolved via discussion with a third member of the review team.

### Outcome measures

#### Dependent variables

##### Primary outcomes

Clinical trials of AN adopt relatively homogenous continuous outcomes, commonly including BMI and global eating disorder psychopathology. The use of dichotomous cut-offs related to BMI outcome are complex, given the wide variation across studies for entry BMI. Therefore, this review will give priority to the use and analysis of continuous variables. Two primary outcomes will be selected: (i) changes to BMI and (ii) changes to global eating disorder psychopathology, measured by using the end point global score on the Eating Disorder Examination (EDE; an investigator-based structured and standardised interview or the self-report questionnaire [17]). If the EDE was not used, we will consider other standardised rating scales. This represents a more relaxed criteria than remission and has been chosen so that we can identify which therapies may be more effective for one outcome than another, given that both do not necessarily improve together over the course of treatment.

##### Secondary outcomes

Given that outpatient treatment for AN varies between 5 and 12 months [13, 14], remission (strict criteria) will be defined as the total number of patients who, at 12-month post-randomization, attained over the previous 28 day period: (i) BMI > 18.5, and (ii) global eating disorder psychopathology or global assessment of functioning or health-related quality of life to within 1 SD of community norms. Use of this definition has shown that patients with AN are indistinguishable from healthy controls on several eating disorder related cognitions [18], and has been used across a number of treatment studies [13–15, 19].

In order to capture improvement that does not meet strict criteria for remission, we will also examine the total number of patients who, at 12-months post-randomisation, attained over the previous 28 day period: (i) an increase in 2 BMI points from baseline, and (ii) a decrease of global eating disorder psychopathology or global assessment of functioning or health-related quality of life to within 2 SD of community norms. Two other secondary outcome measures will be used. The first is drop-out, which indicated acceptability of the treatment. The second is the use of hospitalisation after treatment has been completed over the follow-up period. Studies that do not report data in a format amenable to the NMA will be described in the systematic review.

#### Independent variables

The literature suggests many candidates for effect predictors (variables associated with response regardless of the treatment) and for effect modifiers (variables associated with differential response depending on the treatment) in the treatment of anorexia. We have listed in Table 1 the possible candidate variables for effect predictors and effect modifiers based on the literature [20]. The variables will first be limited by their availability in the included original studies, but when several variables that measure similar things are available, the research team will discuss those we believe are the most important predictors and those that should be included in the model. We will also examine this limited set of variables in the meta-regression for the primary outcomes only.

**Table 1:** Candidate variables for effect predictors and effect modifiers based on the literature

Category of variable	Specific variable and expected direction in terms of predicting better outcome
Life and social history	Less child maltreatment
	Better social adjustment
	Being male
History of present illness	Older age of onset
	Shorter duration of illness
	Less prior treatments
Present illness: symptomatology	Higher baseline body mass index
	Less frequent binge/purge behaviours
	Lower levels of comorbid psychopathology
	Less days of inpatient hospitalization for the purpose of medical stabilization during the treatment trial
	Use of adjunctive treatment over the follow-up period
	Higher number of outpatient sessions

### Data collection and management

Individual participant data including the dependent as well as independent variables as specified below will be sought from the principal investigators of all the identified trials. The veracity of the obtained data will be cross examined by calculating the summary statistics (numbers and percentages, or means and SDs) of the baseline demographic as well as clinical variables, and comparing them against the published reports.

### Data extraction

Two reviewers will then independently read each article/study report, extract the data and assess the quality of the study (see details below). We will design and use a structured data extraction form to ensure consistency of information and appraisal for each study. Information extracted will include study characteristics (such as lead author, publication year, country and journal), participant characteristics (such as diagnostic criteria for AN, age, sex, setting, baseline BMI, concurrent diagnosis of another mental disorder, number of days of inpatient treatment for the purpose of medical stabilisation, number of outpatient sessions, days of adjunctive treatment over the follow-up period), intervention details, and outcome measures. Two review authors will ascertain that the data are entered correctly into the final dataset. When published and unpublished studies provide different values, we will prioritise the unpublished data.

#### Continuous outcomes

We will extract means and SD from each study and enter these into an Excel sheet. In the absence of SD, we will use the guidelines provided by the Cochrane Handbook for Systematic Reviews of Interventions [21] for estimating SD from confidence intervals, standard errors, t values, *P* values, F values, and changes from baseline. We will use comprehensive meta-analysis software (https://www.meta-analysis.com) to estimate the required statistics. When required statistics are not recorded, the authors will be asked to supply the data. If SD are not reported and not provided by the authors, the mean value of known SD will be calculated and borrowed from the group of included studies. Change in both directions will be noted i.e., increases and decreases in the two primary outcome variables, BMI and global eating disorder psychopathology. When mixed method repeated measures or other appropriate imputation methods are used, we will prefer these results.

#### Dichotomous outcomes

We opt for the number of respondents per treatment arm who meet criteria for remission (both strict and lenient criteria). When these numbers are not reported but baseline mean and endpoint mean and standard deviations of the eating disorder rating scales (such as EDE) are provided, we will calculate the number of responding patients at 12-month post-randomization (range 40 to 60 weeks) employing multiple imputation [22]. Below we also discuss our strategy when means and/or standard deviations are not reported in the articles.

#### Missing outcome data

Using the individual patient data, we will judge completion as attending 75% of the allotted sessions. Outcomes of patients that leave the study early are typically imputed by the trialists, and it is very rare for an article to report the outcome separately for fully observed and imputed data and the summary statistics that we will collect are bound to refer to both study completers, moved to a different treatment, and patients who terminated treatment all together. The appropriateness of the imputation method to account for early dropouts will be considered in the Risk of Bias assessment. After imputations at the individual participant level by the original authors, the outcome might be unknown (and not imputed by the original authors) for a very small proportion of study participants. For the dichotomous efficacy outcome we will assume that participants with an unknown outcome are non-responders.

#### Length of trial

Typically across different studies efficacy is assessed at varying time periods, but all studies typically report on an observation around 12-months post-randomisation and this will be our follow up duration of interest. Clinicians need to know whether (and to what extent) treatments sustain effects over a period of time. If 12-month (i.e. 52-week) post-randomisation data are not available, we will use data as close to this as possible (ranging between 40 and 60 weeks).

### Risk of bias assessment

We will assess risk of bias in the included studies using the tool described in the Cochrane Collaboration Handbook as a reference guide [21]. The assessment will be performed by two independent raters. If the raters disagree, the final rating will be made by consensus with the involvement (if necessary) of another member of the review group. We will evaluate the risk of bias in the following domains: generation of allocation sequence, allocation concealment, blinding of study personnel and participants, blinding of outcome assessor, attrition, selective outcome reporting and other domains. Where inadequate details of allocation concealment and other characteristics of trials are provided, the trial authors may be contacted in order to obtain further information. We will not include studies where sequence generation was at high risk of bias and where allocation was clearly not concealed.

### Statistical synthesis of study data

We will synthesise data using a one-step IPD meta-analysis model assuming independent interaction between treatment effects and covariates, as described by Donegan et al. (model 2) [23]. We will ‘borrow strength’ across the multiple time points by assuming that the observations from each patient follow a multivariate normal distribution, thus accounting for the correlation between the observations [12]. Then, for study j comparing treatments X and Y, for the observations at the study’s end point we will assume that:

*m*_*ijX*_ = *u*_*j*_ + *α*_*j*_ *x*_*ij*_, if patient *i* received treatment X

${m}_{ij Y}={u}_j+{\alpha}_j{x}_{ij}+\left({\beta}_{DX}-{\beta}_{DY}\right)\left({x}_{ij}-\overline{x}\right)+{\delta}_{j Y X}+{\mu}_{DX}-{\mu}_{DY}$, if patient *i* received Y

where X is the (arbitrarily chosen) reference treatment for study *j*, *δ*_*j*_ ~ *N*(0, *τ* ^2^) *τ* ^2^ is the heterogeneity (common for all comparisons), *x*_*ij*_ is a covariate, and the coefficients *β* measure the interaction between the relative treatment effects and the covariate values. The coefficients *α*_*j*_ measure the impact of the covariate on the endpoint outcome that is irrespective of the treatment being taken. The model described above pertains to both continuous and dichotomous outcomes. The latter will be assumed to follow a Bernoulli distribution, where *m*_*ijk*_(*k* = *X*, *Y*) will correspond to log-odds. We will opt for IPD data from all included studies; however, if there are studies for which only aggregated data are available (AD) we will include those as described in Donegan et al. [23] by distinguishing within-trial and between-trials (model 5). If a trial is identified that compares all three interventions we will substitute the random-effects distribution of *δ*_*j*_ for its bivariate distribution. The model will be fitted in OpenBUGS using vague priors for all location parameters (effect sizes and regression coefficients). For the heterogeneity, we will use a half-normal prior on the standard deviation. We will use as regressors the select variables from the above list.

We are going to include studies examining both non-pharmacological and pharmacological interventions for outpatient treatment of AN in adults. Of the trials of which we are currently aware [1], there may be no monotherapy studies utilising pharmacology for the treatment of the acute phase of AN (typically medication is used as an adjunctive therapy), so it may be that no common comparator exists between non-pharmacological and pharmacological interventions. In this case we will have two separate networks: one network of studies which compare drug X with drug Y or placebo as an adjunctive therapy (Network 1) and another network of studies comparing psychotherapy A with psychotherapy B or TAU (Network 2) as a monotherapy approach.

#### Missing data

We will impute missing data in OpenBUGS assuming a missing at random (MAR) missingness mechanism [24].

#### Estimation of heterogeneity and inconsistency

We expect that heterogeneity and inconsistency introduced by variability in patient characteristics will be accounted for by the meta-regression model. Residual heterogeneity in the data will be measured by monitoring the common heterogeneity parameter *τ* ^2^ and by comparing it to its empirical distribution [25, 26]. Residual inconsistency will be assessed by estimating the difference *w* between direct and indirect estimates in the drug-psychotherapy-combination loop of evidence. This will be achieved by adding *w* in the equation for *m*_*ijP*_, for studies comparing psychotherapy and combination therapy.

#### Assessment of inconsistency

The strategical and conceptual evaluation of transitivity (i.e., whether it was equally likely that any patient in the network could have been given any of the treatments in the network) will be supplemented with a statistical evaluation of consistency, the agreement between direct and indirect evidence. We will employ local as well as global methods to evaluate consistency [27]. Local methods detect ‘hot spots’ of inconsistency, evidence loops that are inconsistent or comparisons for which direct and indirect evidence disagree. We will employ the loop-specific approach to evaluate inconsistency within each loop of evidence, and a method that separates direct evidence from indirect evidence provided by the entire network [28]. We will also evaluate consistency in the entire network by calculating the I2 for network heterogeneity, inconsistency, and for both [29, 30]. Interpretation of the statistical inference about inconsistency will be carried out with caution and possible sources of inconsistency will be explored even in the absence of evidence for inconsistency.

#### GRADE quality assessment of all comparisons in the network

We will also assess the quality of evidence contributing to network estimates of the main outcomes with the GRADE framework, which characterizes the quality of a body of evidence on the basis of the study limitations, imprecision, inconsistency, indirectness and publication bias [27]. The starting point for confidence in each network estimate is high, but will be downgraded according to the assessments of these five domains.

#### Dissemination

We will publish findings from this systematic review in a peer reviewed scientific journal and dataset will be made freely available. The completed review will be disseminated electronically, in print and on social media, where appropriate.

## Discussion

Our aim to is examine individual data across all available outpatient studies of adult AN in order to offer up a more definitive comparison between different treatments, as well as examining the impact of moderators of treatments, in order to make specific conclusions about which therapies may best suit which patients. Recent eating disorder practice guidelines for Australia and New Zealand [31] concluded that we are unable to make any robust and direct comparison between commonly used psychological treatments for AN, like CBT, interpersonal psychotherapy, or psychodynamic psychotherapy, and that interpretation of findings where specific psychological therapies are compared to other therapies is problematic because of methodological problems. The guidelines also note that currently that CBT and its many forms is probably the most recommended specific psychotherapy for AN.

This proposed NMA has the potential to resolve this impasse, by having greater power to directly compare therapies to each other, in part due to the emergence of many new studies since the last meta-analysis was published, and in part due to the using the network to compare therapies that have not previously been directly compared to each other. This will allow us to start to make some conclusions as to whether the evidence supports some front runner therapies for adult outpatient AN. Given the current state of affairs, which seems to support the idea that a range of different therapies may have merit [31], the NMA can use individual data to examine putative moderators of treatment, and provide a more nuanced suggestions for clinicians, such as which type of therapy works best for which type of patient.

## Appendix: List of research registers that will be searched

- ◦ European Clinical Trials Database (EudraCT)
- ◦ UMIN Clinical Trials Registry (UMIN-CTR)
- ◦ JapicCTI (http://www.clinicaltrials.jp/user/cteSearch_e.jsp)
- ◦ Japan Medical Association Centre for Clinical Trials (JMACCT)
- ◦ UK National Research Register [for archived content only]
- ◦ WHO International Clinical Trials Registry Platform (WHO ICTRP) [includes datasets from the following providers]:

o Australian New Zealand Clinical Trials Registry (ANZCTR)

o Brazilian Clinical Trials Registry (ReBec)

o Chinese Clinical Trial Registry (ChiCTR)

o Clinical Research Information Service - Republic of Korea (CRIS)

o Clinical Trials Registry - India CTRI)

o ClinicalTrials.gov

o Cuban Public Registry of Clinical Trials (RPCEC)

o EU Clinical Trials Register (EU-CTR)

o German Clinical Trials Register (GermanCTR)

o ISRCTN

o Iranian Registry of Clinical Trials (IRCT)

o Japan Primary Registries Network (JPRN)

o Netherlands National Trial Register (NTR)

o Pan African Clinical Trial Registry (PACTR)

o Sri Lanka Clinical Trials Registry (SLCTR)

o Thai Clinical Trials Register (TCTR)

For extra sensitivity we will undertake our own searches in some of the key registers that provide datasets for inclusion in WHO ICTRP. The list of registers is as follows:

- Australian Clinical Trials Registry (ANZCTR)
- clinicaltrials.gov
- ISRCTN
- Netherlands National Trial Register (NTR)

## Abbreviations

AN: Anorexia nervosa
BMI: Body mass index
CBT: Cognitive behaviour therapy
CrI: Credible Intervals
DSM: Diagnostic and Statistical Manual
ICD: International Classification of Diseases
MANTRA: Maudsley Model of Anorexia Nervosa Treatment for Adults
ORs: Odds ratio
RCTs: Randomised controlled trials
SD: Standard deviation
SMD: Standardized mean differences
